# Efficacy and immunogenic effects of Tumor Treating Fields (TTFields) in preclinical models of pancreatic ductal adenocarcinoma, with and without gemcitabine/nab‐paclitaxel

**DOI:** 10.1002/ijc.70408

**Published:** 2026-02-27

**Authors:** Tal Kan, Tharwat Haj Khalil, Yiftah Barsheshet, Tali Voloshin, Lilach Koren, Bella Koltun, Cfir David, Kerem Wainer‐Katsir, Avital Vorontsova, Boris Brant, Simona Zisman‐Rozen, Hila M. Ene, Roni Frechtel‐Gerzi, Shay Cahal, Anat Klein‐Goldberg, Lena Lifshitz, Efrat Zemer Tov, Mai Shai, Adi Haber, Moshe Giladi, Uri Weinberg, Yoram Palti

**Affiliations:** ^1^ Novocure Ltd. Haifa Israel; ^2^ Novocure GmbH Baar Switzerland

**Keywords:** c‐Myc, gemcitabine, nab‐paclitaxel, pancreatic ductal adenocarcinoma (PDAC), Tumor Treating Fields (TTFields)

## Abstract

Tumor Treating Fields (TTFields) are an approved cancer therapy for glioblastoma (GBM), pleural mesothelioma, and non‐small cell lung cancer (NSCLC). A recent phase 3 trial of TTFields therapy concomitant with standard‐of‐care gemcitabine and nab‐paclitaxel (Gem/NabP) as a first‐line treatment for unresectable, locally advanced pancreatic adenocarcinoma demonstrated a significant increase in overall survival. The current study evaluated the effects of TTFields in preclinical pancreatic ductal adenocarcinoma (PDAC) models. The vast majority of PDAC patients harbor KRAS mutations, which are associated with a more aggressive disease phenotype and increased therapy resistance, driven in part by overexpression of the key transcription factor c‐Myc. In the current study, TTFields application significantly suppressed c‐Myc expression and induced immunogenic cell death (ICD)—characterized by increased calreticulin cell‐surface exposure, extracellular ATP secretion, and elevated HMGB1 release—in pancreatic cancer models. These effects were further enhanced when TTFields were applied concomitantly with Gem/NabP. Causality between c‐Myc modulation and immune readouts was not established. In vivo, TTFields application induced a systemic immune response, evidenced by dendritic cell activation, increased effector memory T cells, and greater tumor leukocyte infiltration. TTFields concomitant with Gem/NabP significantly reduced tumor volume, decreased tumor monocytic myeloid‐derived suppressor cells (M‐MDSC), and increased the tumor lymphocyte‐to‐monocyte ratio (LMR) compared to all other treatment groups. These findings support the potential of TTFields to enhance therapeutic efficacy. Moreover, TTFields‐induced tumor immunogenicity may enable combination strategies with immunotherapies. A phase 2 clinical trial investigating TTFields with Gem/NabP and immune checkpoint inhibitors (ICIs) for metastatic PDAC is currently underway.

## Graphical abstract

What's new?

Adaptive immune responses are typically limited in pancreatic ductal adenocarcinoma, resulting in minimal efficacy of immune checkpoint inhibitors. Tumor Treating Fields (TTFields) therapy, which uses low‐intensity intermediate‐frequency electric fields to disrupt cancer cell processes, has been approved for certain cancers. This study found that TTFields downregulated c‐Myc expression and enhanced cytotoxicity and immunogenic cell death in pancreatic ductal adenocarcinoma cell lines. In mice, TTFields induced systemic immune activation and, when delivered together with gemcitabine/nab‐paclitaxel, significantly reduced tumor volume. These findings provide a strong rationale for future investigations into the integration of TTFields with chemotherapy and immunotherapy strategies in pancreatic ductal adenocarcinoma.

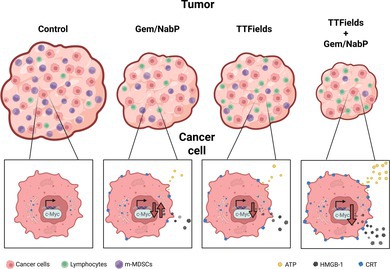

**Abbreviations**
7‐AAD: 7‐aminoactinomycin D
AnnV: annexin V
APCs: antigen‐presenting cells
ATCC: American Type Culture Collection
BLI: bioluminescence imaging
cDNA: complementary DNA
CRT: calreticulin
DCs: dendritic cells
eIF2α: eukaryotic translation initiation factor 2α
FDR: false discovery rate
FOLFIRINOX: fluorouracil, irinotecan and oxaliplatin
GBM: glioblastoma
GSEA: gene set enrichment analysis
HMGB1: high‐mobility group box 1
HRP: horseradish peroxidase
IC_50_
: half maximal inhibitory concentration
ICD: immunogenic cell death
ICIs: immune checkpoint inhibitors
LMR: lymphocyte to monocyte ratio
MHC‐II: major histocompatibility complex class II
M‐MDSCs: monocytic myeloid‐derived suppressor cells
NabP: nab‐paclitaxel
NES: normalized enrichment score
NSCLC: non‐small cell lung cancer
PBMCs: peripheral blood mononuclear cells
PCA: principal component analysis
PDAC: pancreatic ductal adenocarcinoma
QA: quinacrine dihydrochloride
qPCR: quantitative polymerase chain reaction
SD: standard deviation
SEM: standard error of the mean
STR: short tandem repeat
TME: tumor microenvironment
TTFields: Tumor Treating Fields

## 1 INTRODUCTION

Pancreatic cancer remains a major global health challenge. In the United States, it is currently the third leading cause of cancer‐related death and is projected to become the second leading cause by 2030,^1^, ^2^ highlighting the urgent need for innovative treatments.

Pancreatic ductal adenocarcinoma (PDAC) accounts for approximately 92% of all pancreatic cancers.^1^, ^2^ Most patients present with either locally advanced or metastatic disease, with median survivals of 16 and 8 months, respectively.^1^ Despite the use of intensive chemotherapy regimens such as FOLFIRINOX or gemcitabine combined with nab‐paclitaxel (Gem/NabP), treatment outcomes remain poor.^1^, ^2^

KRAS mutations occur in approximately 90% of PDAC cases and are associated with aggressive disease and poor prognosis.^3^ These mutations indirectly drive overexpression of c‐Myc, a master regulator of cellular growth and metabolism, thereby contributing significantly to progression and therapeutic resistance.^3^, ^4^ Both KRAS and c‐Myc play critical, interconnected roles in disease aggressiveness and resistance to current therapies.^5^ Recently, KRAS inhibitors targeting the G12C subtype were approved for treatment of non‐small cell lung cancer (NSCLC) and colorectal cancer.^6^ However, these inhibitors have very limited applicability in PDAC, as the KRAS G12C mutation subtype occurs in only about 1% of PDAC patients.^7^

Immune checkpoint inhibitors (ICIs) have transformed cancer therapy, yet PDAC is typically an immune‐excluded tumor,^8^, ^9^ characterized by low levels of tumor‐infiltrating lymphocytes, an immunosuppressive tumor microenvironment (TME), and a limited neoantigen load, consistent with PDAC's relatively low tumor mutational burdan. PDAC typically presents ~35–38 predicted neoantigens per tumor, markedly fewer than more immunogenic cancers such as lung cancer (~112) or melanoma (~370), although most PDACs still harbor potentially targetable neoantigens.^10^, ^11^ These features collectively limit immune recognition and adaptive immune responses, resulting in minimal efficacy of ICIs in PDAC.

Tumor Treating Fields (TTFields) therapy is a cancer treatment utilizing low‐intensity intermediate‐frequency electric fields that exert physical forces to disrupt cancer cell processes.^12^ By disrupting microtubule polymerization and septin organization, TTFields induce abnormal chromosome segregation, ER stress, and aneuploidy.^13^, ^14^ This in turn leads to immunogenic cell death (ICD) and activation of a systemic anti‐tumor immune response.^15^, ^16^ Currently, TTFields therapy is approved for treatment of newly diagnosed and recurrent glioblastoma (GBM), pleural mesothelioma, and NSCLC. TTFields therapy is applied to patients non‐invasively and locoregionally, with a portable fields generator and arrays attached to the skin around the tumor location. TTFields have demonstrated preclinical efficacy in pancreatic cancer models and improved survival when used together with Gem/NabP in a recent phase 3 clinical trial (PANOVA‐3, NCT03377491).^17^

The present study evaluates the preclinical efficacy of TTFields with Gem/NabP in PDAC.

## 2 METHODS

### 2.1 Cell culture

Human AsPC‐1 (RRID: CVCL_0152) and BxPC‐3 (RRID: CVCL_0186) PDAC cell lines were obtained from the American Type Culture Collection (ATCC). Murine Panc02 PDAC cell line expressing luciferase (RRID: CVCL_A8QL) was obtained from GenTarget Inc. The cells were grown in RPMI‐1640 medium (30‐2001, ATCC) supplemented with 10% (v/v) fetal bovine serum (10270106, Gibco) and 50 μg/mL penicillin–streptomycin (L0022, Biowest) in a 37°C humidified incubator supplied with 5% CO_2_. All cell lines were authenticated using short tandem repeat (STR) profiling within the last 3 years. Cell lines were tested for mycoplasma contamination using MycoStrips™ (InvivoGen, Cat. No. rep‐mys1) prior to cell bank cryopreservation, and monthly for thawed cells.

### 2.2 TTFields in vitro experiments

AsPC‐1, BxPC‐3, and Panc02‐Luc cell suspensions (8 × 10^4^, 16 × 10^3^, and 10^4^ cells/mL, respectively) were seeded onto 22 mm coverslips in inovitro dishes (Novocure), incubated overnight, and 2 mL fresh media was added, with or without Gem (173242, Novartis), and NabP (107428001, Bristol Myers Squibb) at concentrations identified as the combined IC50 for each cell line (2.18 nM Gem/0.77 nM NabP for AsPC‐1; 0.0325 nM Gem/0.0114 nM NabP for BxPC‐1; and 4.91 nM Gem/1.72 nM NabP for Panc02). The dishes were then connected to the inovitro system (Novocure) and TTFields (1.7 V/cm RMS) were applied for 24, 48, or 72 h at a frequency of 150 kHz, as previously described.^18^

### 2.3 RNA‐seq analysis

RNA purification, library preparation, sequencing, alignment, and differential expression were performed as previously described.^19^ The sequencing coverage and quality statistics for each sample are summarized in Table S1, Supporting Information. Gene set enrichment was performed with GSEA software using the MsigDB hallmarks gene set.^20^

### 2.4 Quantitative polymerase chain reaction

RNA was isolated (48300, Norgen Biotek) and cDNA was synthesized (95047–200, Quantabio) according to the manufacturer's instructions. Quantitative polymerase chain reaction (qPCR) was carried out with the qPCRBIO SyGreen blue mix (PB20.15‐05, PCR Biosystems) on a QuantaStudio 1 thermocycler (Applied Biosystems), utilizing primers for c‐Myc: Forward 5′‐TGGTGCTCCATGAGGAGAC‐3′; Reverse 5′‐CAGACTCTGACCTTTTGCCA‐3′. UBC was used for normalization: Forward 5′‐CTGGAAGATGGTCGTACCCTG‐3′; Reverse 5′‐GGTCTTGCCAGTGAGTGTCT‐3′.

### 2.5 Immunoblotting

Cells were washed with PBS and lysed with RIPA buffer (R0278, Sigma‐Aldrich). Thirty microgram of protein samples were resolved by SDS‐polyacrylamide gel electrophoresis under reducing conditions and transferred to polyvinylidene difluoride membranes. Membranes were probed with primary antibodies: eIF2α (CST‐9722S, Cell Signaling), phosphorylated eIF2α (CST‐9721S, Cell Signaling), c‐Myc (AB32, Abcam), and vinculin (V9131, Sigma‐Aldrich). Secondary horseradish peroxidase (HRP)‐conjugated goat anti‐mouse or anti‐rabbit antibody (AB97023 or AB6721, Abcam) and a chemiluminescent substrate (WBLUF, Merck) were used for visualization. Signals were recorded on the GeneGnome XRQ gel imager (AlphMetrix Bitech). Densitometric readings were normalized to vinculin with ImageJ software (NIH) and expressed as fold change relative to control.

### 2.6 Cell count

Cells were counted using Cytek® Northern Lights (Cytek® Biosciences, Fremont, CA) flow cytometer, and cell count presented as the percentage relative to control.

### 2.7 Apoptosis

Cells were stained with fluorescein isothiocyanate (FITC)‐conjugated Annexin V (AnnV) and 7‐Aminoactinomycin D (7‐AAD) as per manufacturer's instructions (640945 and 420404, BioLegend). Cells were acquired by flow cytometry, and percentages of live (AnnV^−^/7AAD^−^), apoptotic (AnnV+/7AAD^−^), and dead (AnnV+/7AAD+) cells were quantified.

### 2.8 Calreticulin exposure

Cells were stained with rabbit anti‐mouse calreticulin (CRT) antibody (AB2907, Abcam) followed by donkey anti‐rabbit Alexa Fluor 488 conjugated antibody (AB150073, Abcam) and 7‐AAD. Cells were acquired by flow cytometry, and the percentage of live cells positive to CRT (CRT^+^/7AAD^−^) was quantified.

### 2.9 ATP intracellular depletion

Cells were incubated with 0.05 μM quinacrine dihydrochloride (QA; Q3251, Sigma‐Aldrich) and 7‐AAD. Cells were acquired by flow cytometry, and the percentage of live cells negative to QA (QA^−^/7AAD^−^) was quantified.

### 2.10 Detection of HMGB1

Supernatants were collected and were used for quantification of HMGB1 by HTRF assay (62HMGPEG, Waltham) according to the manufacturer's instructions.

### 2.11 Animal study design

Animal studies were approved by the Animal Experimentation Committee according to the Ministry of Health by the Israel National Council for Animal Experimentation (approval number: NPC‐No‐IL‐2308‐451‐4) and performed in accordance with guidelines and regulations. Male C57Bl/6 mice (Harlan Laboratories), 10‐weeks of age, were inoculated into the pancreas with 4 × 10^5^ luciferase‐expressing Panc02 cells (15 μL in 1:1 RPMI:matrigel). A 1.5‐cm incision was made to expose the pancreas and inject the cells into the distal portion of the pancreas, as previously described.^21^ Tumor bioluminescence (BLI) was measured using the IVIS Lumina III (PerkinElmer) 13 days after cell injection, and mice were randomized into four groups (balanced according to tumor total flux): (1) sham (heat), vehicle (saline); (2) TTFields, vehicle; (3) sham, Gem/NabP; and (4) TTFields, Gem/NabP. TTFields (150 kHz, 4.4–5.6 V/cm RMS) or heat were applied to mice torsos using the inovivo system (Novocure), continuously for 10 days, starting from day 14 of inoculation. Gem/NabP (50 mg/kg each) or vehicle were injected intraperitoneally on days 14 and 21 of inoculation. At treatment end (day 24) the mice were anesthetized, blood was obtained by submandibular bleeding, and tumor BLI signal was recorded. Then, the mice were euthanized by cervical dislocation. Tumors were dissected, measured with Vernier calipers, and their volume calculated using the formula width^2^ × length × 0.5. Blood and tumors were then processed as previously described.^15^

### 2.12 Blood/tumor immunoprofiling

Tumor single cell suspensions and blood samples were stained with Zombie NIR (423106, BioLegend), incubated with mouse Fc block (156604, BioLegend; anti‐CD16/CD32), and then stained with the following fluorochrome‐conjugated anti‐mouse antibodies: CD45‐AF700 (103128, BioLegend), CD11b‐PerCP (101230, BioLegend), CD3e‐BV750 (100249, BioLegend), CD4‐BV605 (100451, BioLegend), CD8a‐PE‐Fire 700 (100792, BioLegend), Ly6G‐BV650 (127641, BioLegend), Ly6C‐BV570 (128029, BioLegend), CD11c‐PE/Dazzle 594 (117348, BioLegend), CD44‐Alexa Fluor 532 (58‐0441‐82, Invitrogen), CD62L‐BV480 (746726, BD Biosciences), CD19‐PE/Fire 640 (115574, BioLegend), CD49b‐Pacific blue (108918, BioLegend), NK1.1/APC‐Cy7 (108724, BioLegend), and MHC class II (MHC‐II)‐BV510 (107635, BioLegend). Cyto‐Fast™ Fix/Perm Buffer Set (426803, BioLegend) was utilized according to manufacturer instructions. Data was acquired on Cytek® Northern Lights flow cytometer and analyzed using FlowJo software.

### 2.13 Statistical analysis

Data were analyzed with GraphPad Prism software. Normality was tested with Shapiro–Wilk test, and statistical significance was assessed using a parametric or nonparametric test as appropriate. *p*‐values ≤.05 were considered statistically significant and were indicated as *p < .05; **p < .01; and ***p < .001.

## 3 RESULTS

### 3.1 TTFields downregulate the expression of the master regulator c‐Myc and associated downstream pathways in PDAC cells

**FIGURE 1:**
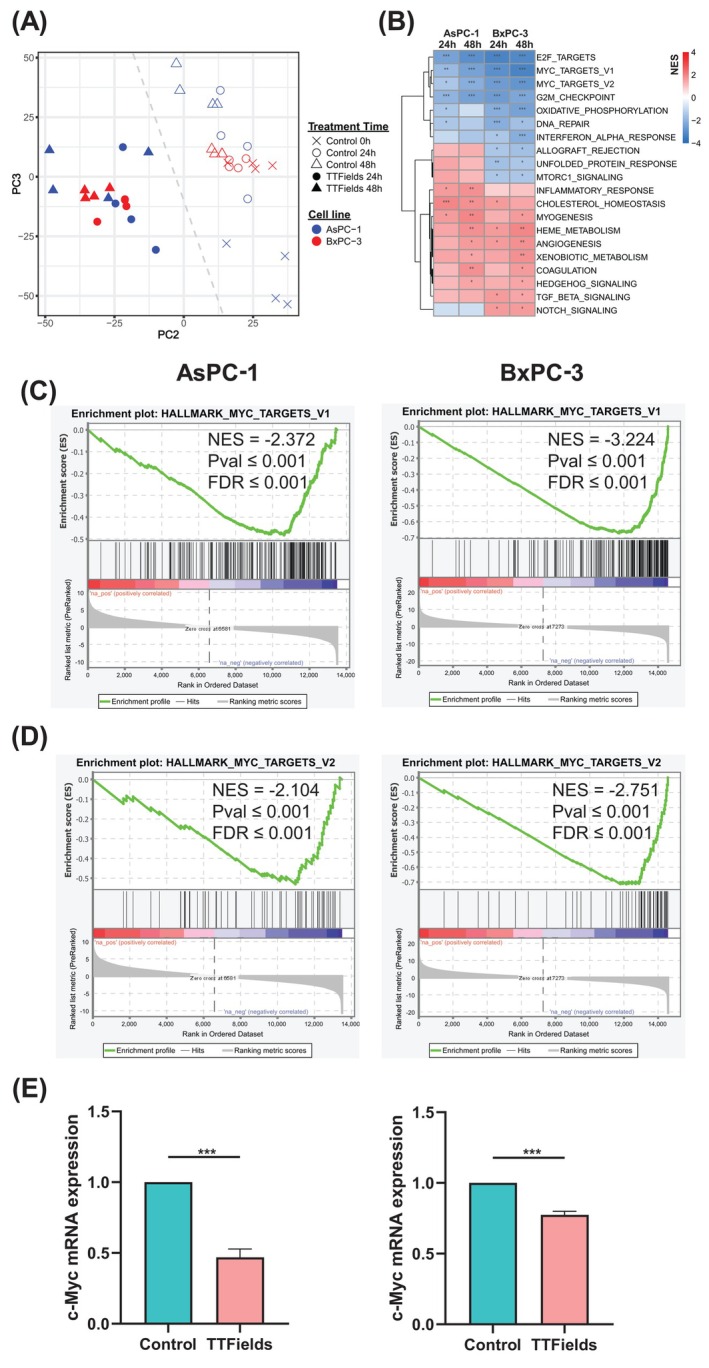
TTFields downregulate the expression of the master regulator c‐Myc and associated downstream pathways in PDAC cells. Human PDAC cell lines AsPC‐1 and BxPC‐3 were treated with 150 kHz TTFields and subjected to transcriptomics analysis. (A) Principal component analysis (PCA) of global gene expression profiles for control cells and those treated with TTFields for 24 or 48 h. (B) Heatmap generated through Gene Set Enrichment Analysis (GSEA) using MsigDB hallmark gene sets, displaying normalized enrichment scores (NES) for significantly altered pathways common across at least two samples (FDR ≤0.05). Statistical significance levels are indicated as *FDR <0.05, **FDR <0.01, and ***FDR <0.001. (C, D) GSEA analysis of Myc targets V1 and Myc targets V2 MsigDB hallmark pathways after 48‐h TTFields treatment compared to untreated controls. (E) Real‐time quantitative PCR validation of c‐Myc mRNA levels following 72‐h TTFields treatment versus untreated controls, normalized to the housekeeping gene UBC. Data represent mean ± SEM; statistical significance (***p < .001) was calculated using Student's *t* test.

To identify targets affected by TTFields treatment in PDAC cells, RNA sequencing was performed on AsPC‐1 and BxPC‐3 cells following 24 or 48 h of TTFields exposure. Principal component analysis (PCA) revealed distinct clustering between control and TTFields‐treated cells for both cell lines (Figure 1A), indicative of substantial TTFields‐induced alterations in gene expression. Gene Set Enrichment Analysis (GSEA) using the MsigDB hallmark gene sets identified Myc targets V1 and V2, E2F targets, and G2M checkpoint pathways as the most significantly downregulated pathways (Figure 1B). Individual GSEA enrichment plots for Myc targets V1 and V2 validate and visualize the strength of enrichment for Myc‐regulated genes following TTFields application (Figure 1C,D). To determine whether downregulation of Myc‐related pathways was mediated by changes in expression of their upstream regulator c‐Myc, quantitative PCR (qPCR) was conducted. Indeed, c‐Myc RNA levels were significantly reduced in both cell lines following 72 h of TTFields application (Figure 1E).

### 3.2 Concomitant treatment with TTFields and Gem/NabP enhances cytotoxicity and promotes ICD in PDAC cells

**FIGURE 2:**
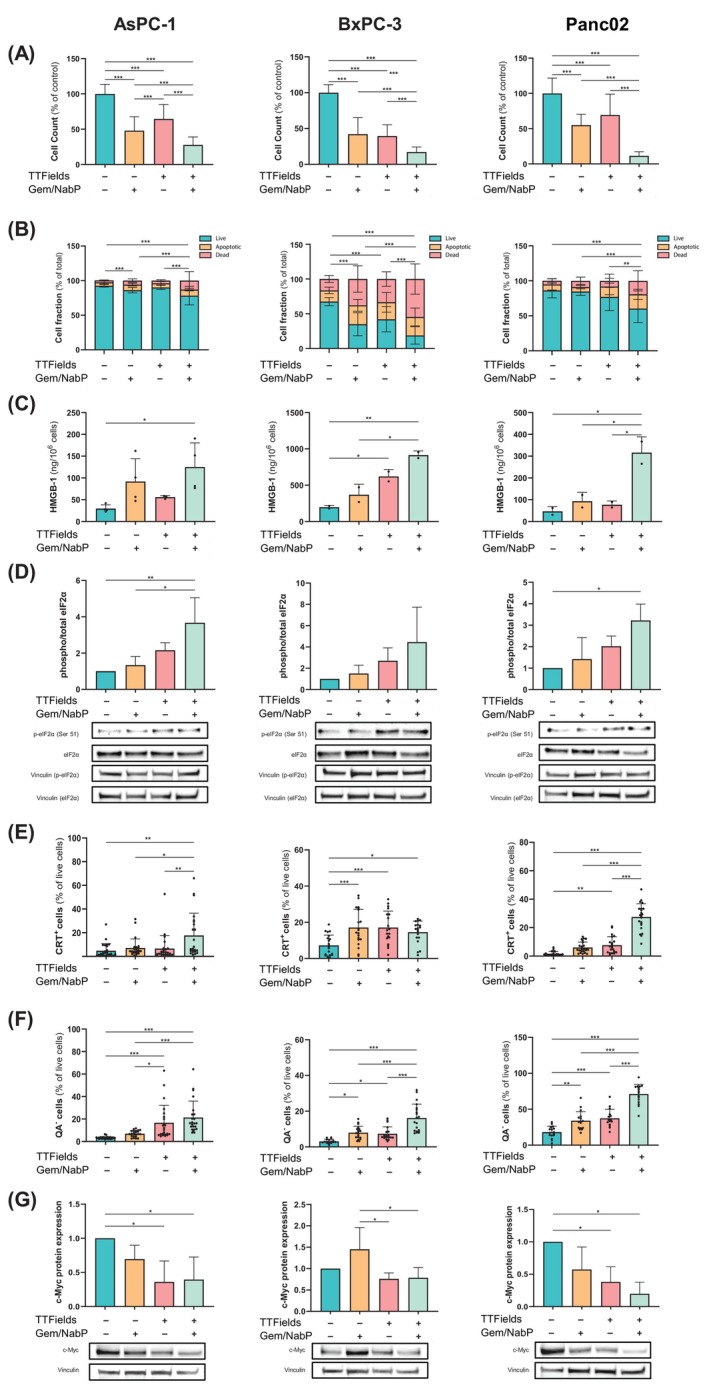
Concomitant treatment with TTFields and Gem/NabP enhances cytotoxicity and promotes immunogenic cell death in PDAC cells. Human PDAC cell lines AsPC‐1 and BxPC‐3, along with murine Panc02 cells, were treated with 150 kHz TTFields and/or Gem/NabP (at concentrations with an effect of IC50 for the combination: 2.18 nM Gem/0.77 nM NabP for AsPC‐1; 0.0325 nM Gem/0.0114 nM NabP for BxPC‐1; and 4.91 nM Gem/1.72 nM NabP for Panc02) for 72 h. Efficacy was assessed by measuring: (A) cell counts and (B) proportions of live (AnnV^−^/7AAD^−^), apoptotic (AnnV+/7AAD^−^), and dead (AnnV+/7AAD+) cells. Immunogenic cell death (ICD) was examined by measurement of: (C) release of HMGB1, (D) phosphorylation levels of eIF2α, (E) exposure of calreticulin on the surface of viable cells (CRT+/7AAD^−^), (F) intracellular ATP depletion in viable cells (QA^−^/7AAD^−^), and (G) c‐Myc protein levels post‐treatment, normalized to vinculin. For Panc02 cells, eIF2A and c‐myc were probed from the same blotting membrane and hence have the same vinculin bands. Results represent mean ± SD *p < .05, **p < .01, and ***p < .001; one‐way ANOVA followed by Tukey's post hoc test; two‐way ANOVA followed by Tukey's post hoc test for apoptosis data, specifically marking differences within the live cell fraction.

We next evaluated the efficacy of 72‐h TTFields treatment, alone or concomitantly with Gem/NabP, in human PDAC cell lines AsPC‐1 and BxPC‐3, as well as in the murine Panc02 cell line. While treatment with the Gem/NabP regimen or TTFields alone reduced cell count (Figure 2A) and percentage of viable cells (Figure 2B) relative to control, these effects were significantly enhanced when TTFields were applied concomitantly with Gem/NabP.

TTFields have previously been shown to induce ICD in GBM and NSCLC models.^15^, ^16^ To evaluate ICD induction in PDAC cells, we measured several hallmark features of ICD: release into the culture supernatant of high‐mobility group box 1 (HMGB1), a molecule that promotes the maturation of antigen‐presenting cells (APCs) (Figure 2C); phosphorylation of eukaryotic translation initiation factor 2α (eIF2α), a marker of endoplasmic reticulum (ER) stress associated with ICD (Figure 2D); calreticulin cell‐surface exposure, known to facilitate antigen uptake by APCs (Figure 2E); and extracellular ATP secretion, which acts as a chemoattractant for APCs (Figure 2F). While Gem/NabP regimen and TTFields alone individually elevated these ICD markers compared to controls, their concomitant application significantly enhanced these effects.

To determine whether these effects were associated with changes in c‐Myc expression, we assessed c‐Myc protein levels following each treatment condition (Figure 2G). The Gem/NabP regimen led to a modest reduction in c‐Myc, whereas TTFields alone induced a more pronounced decrease. Concomitant treatment with Gem/NabP and TTFields did not further decrease c‐Myc protein relative to TTFields alone within the tested time window. Accordingly, we do not infer a mechanistic link between c‐Myc changes and the immune readouts reported in Figure 2C–F.

### 3.3 Concomitant TTFields and Gem/NabP treatment significantly reduces tumor volume and modulates tumor‐associated and systemic immune profiles in an orthotopic PDAC mouse model

**FIGURE 3:**
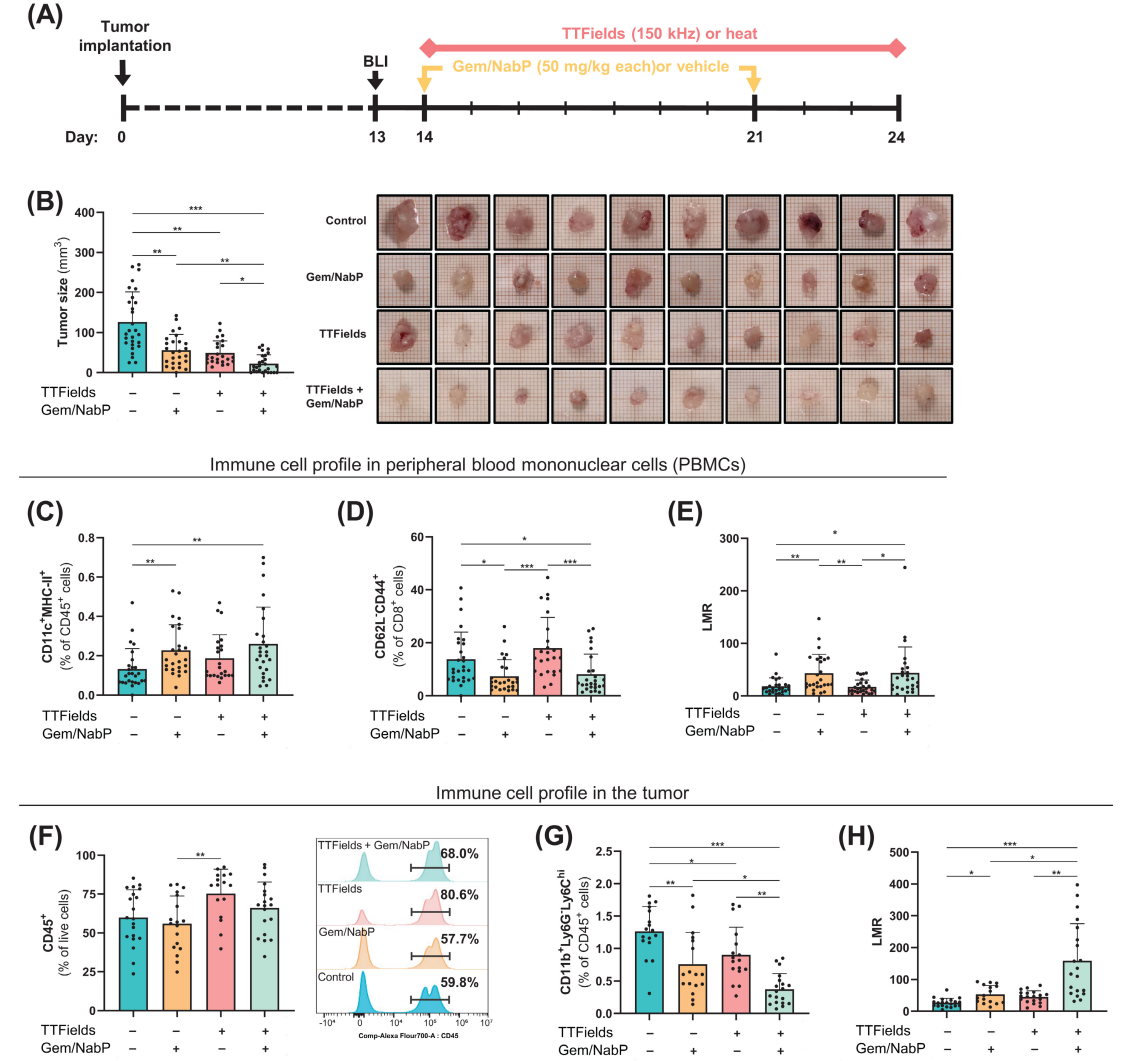
Concomitant TTFields and Gem/NabP treatment significantly reduces tumor volume and modulates tumor‐associated and systemic immune profiles in an orthotopic PDAC mouse model. Orthotopic pancreatic tumors were established in mice by Panc02 cell implantation. Beginning 14 days post‐implantation, mice received continuous 150 kHz TTFields or sham (heat) treatment for 10 consecutive days, with intraperitoneal administration of Gem/NabP (50 mg/kg each) on days 14 and 21 (illustrated in (A). (B) Treatment effects were assessed at study endpoint by measuring tumor volumes. Immune cell profiles in peripheral blood mononuclear cells (PBMCs) were analyzed by flow cytometry, quantifying changes in (C) dendritic cell frequency (CD11c^+^MHC‐II^+^/CD45^+^), (D) effector memory cytotoxic T cells (CD62L^−^CD44^+^/CD8^+^), and (E) lymphocyte‐to‐monocyte ratio (LMR). Additionally, tumor‐infiltrating leukocytes were characterized, assessing (F) total leukocyte infiltration (CD45^+^ cells), (G) monocytic myeloid‐derived suppressor cells (CD11b^+^Ly6G^−^Ly6C^hi^/CD45^+^), and (H) tumor LMR. Results represent mean ± SD; statistical significance marked as *p < .05, **p < .01, and ***p < .001, calculated using one‐way ANOVA with Tukey's post hoc tests or Kruskal–Wallis tests followed by Dunn's multiple comparisons, as appropriate.

We next evaluated the antitumor efficacy of TTFields alone or concomitantly with Gem/NabP using an orthotopic PDAC mouse model. Mice were continuously treated with TTFields or sham (heat) for 10 days, with Gem/NabP or vehicle administered twice during this period (Figure 3A). Endpoint analyses demonstrated that Gem/NabP and TTFields independently exhibited antitumor activity; however, the concomitant treatment significantly enhanced tumor control compared to either treatment alone (Figure 3B). Immunoprofiling of peripheral blood mononuclear cells (PBMCs) revealed an increased frequency of activated dendritic cells (DCs) following Gem/NabP or TTFields, with a significant additional increase observed in the concomitant treatment group (Figure 3C). While total CD8^+^ and CD4^+^ T cell populations remained unchanged (data not shown), TTFields monotherapy increased the frequency of effector memory CD8^+^ T cells. Notably, this increase was attenuated when TTFields were administered concomitantly with Gem/NabP, resulting in lower levels compared to TTFields alone (Figure 3D). Additionally, treatment with Gem/NabP alone or concomitantly with TTFields significantly increased the lymphocyte‐to‐monocyte ratio (LMR) compared to control or TTFields alone (Figure 3E).

Mononuclear cell populations were further analyzed within tumor tissue. Total tumor leukocytes were increased in response to TTFields and TTFields with Gem/NabP, although the latter did not reach statistical significance (Figure 3F). Notably, a significant reduction in monocytic myeloid‐derived suppressor cells (M‐MDSCs; Figure 3G) and an increase in tumor LMR (Figure 3H) were detected in tumors treated with TTFields concomitant with Gem/NabP.

## 4 DISCUSSION

This study demonstrates that TTFields, either alone or concomitantly with Gem/NabP, enhance anti‐tumor efficacy in preclinical PDAC models by promoting cancer cell death and reducing tumor growth, thus supporting the positive outcomes observed in the PANOVA‐3 clinical trial (NCT03377491).^17^ Elevated c‐Myc expression has previously been associated with resistance to Gem and NabP, whereas depletion of c‐Myc has been shown to restore sensitivity to these chemotherapies.^22^, ^23^ While TTFields consistently suppressed c‐Myc at the transcript and protein levels, the immunologic effects of TTFields with Gem/NabP were observed without additional c‐Myc suppression. We therefore view c‐Myc regulation as one of several TTFields‐induced effects that may contribute to chemosensitivity, rather than the key modulatory factor for these immune changes; direct tests of this hypothesis are needed. Furthermore, the mechanism of c‐Myc downregulation by TTFields remains to be defined and is the subject of an ongoing follow‐up study.

Additionally, TTFields induce ICD in PDAC cells, as evidenced in vitro by increased HMGB1 release, calreticulin exposure, and extracellular ATP secretion. Systemic immune activation following concomitant treatment with TTFields and Gem/NabP was demonstrated by increased frequency of activated dendritic cells and elevated LMR in peripheral blood. Within the TME, the concomitant treatment reduced M‐MDSCs and increased the LMR, consistent with previous studies in other cancer models demonstrating that TTFields promote a favorable anti‐tumor immune milieu.^15^, ^16^, ^24^, ^25^ The increased tumor LMR observed following concomitant treatment with TTFields and Gem/NabP suggests a potentially favorable prognostic outcome, aligning with prior clinical evidence.^26^ Furthermore, the observed reduction of M‐MDSCs within the TME implies a diminished immunosuppressive state. Given that PDAC remains largely resistant to ICIs,^8^, ^9^ the current findings support exploring concurrent treatment of ICIs with TTFields, an approach that has demonstrated clinical benefit in metastatic NSCLC.^27^ Future preclinical studies should assess the efficacy of concurrent administration of TTFields with chemotherapy and immunotherapy regimens. Indeed, such therapeutic strategies are currently under clinical investigation in the ongoing phase 2 PANOVA‐4 trial (NCT06390059). Furthermore, additional opportunities for concomitant treatments are supported by RNA sequencing analyses from the present study, demonstrating significant TTFields‐induced downregulation of DNA repair pathways in PDAC cells. These findings align with previous reports in other tumor types, suggesting potential synergy between TTFields and DNA‐damaging agents or inhibitors of DNA repair pathways.^28^, ^29^ This approach merits further exploration in PDAC, particularly with regard to strategies involving PARP inhibitors and FOLFIRINOX. Currently, PARP inhibitors are approved exclusively for metastatic PDAC patients harboring germline BRCA mutations, a subgroup comprising only approximately 5% of the patient population.^7^ By potentially inducing a “BRCAness” phenotype, TTFields treatment might broaden the applicability of PARP inhibitors to a larger subset of PDAC patients.

In summary, this study demonstrates that TTFields have the potential to enhance the therapeutic efficacy of standard‐of‐care chemotherapy for PDAC by downregulating c‐Myc expression, inducing ICD, and reshaping the tumor immune microenvironment towards a more immune‐inflamed phenotype. These findings provide a strong rationale for future investigations into the integration of TTFields with chemotherapy and immunotherapy strategies in PDAC.

## AUTHOR CONTRIBUTIONS

**Tal Kan:** Conceptualization; data curation; formal analysis; investigation; methodology; project administration; visualization; validation; writing – review and editing. **Tharwat Haj Khalil:** Data curation; formal analysis; investigation. **Yiftah Barsheshet:** Conceptualization; data curation; formal analysis; methodology; investigation; project administration; supervision; validation. **Tali Voloshin:** Conceptualization; data curation; formal analysis; investigation; methodology; project administration; supervision; validation; visualization; writing – review and editing; writing – original draft. **Lilach Koren:** Data curation; investigation; writing – original draft. **Bella Koltun:** Data curation; investigation. **Cfir David:** Data curation; investigation. **Kerem Wainer‐Katsir:** Data curation; software; formal analysis; visualization. **Avital Vorontsova:** Data curation; investigation. **Boris Brant:** Data curation; investigation. **Simona Zisman‐Rozen:** Data curation; investigation. **Hila M. Ene:** Data curation; investigation. **Roni Frechtel‐Gerzi:** Data curation; investigation. **Shay Cahal:** Data curation; investigation; methodology. **Anat Klein‐Goldberg:** Data curation; investigation. **Lena Lifshitz:** Data curation; investigation. **Efrat Zemer Tov:** Data curation; investigation. **Mai Shai:** Investigation; data curation. **Adi Haber:** Formal analysis; visualization; writing – original draft; writing – review and editing. **Moshe Giladi:** Conceptualization; project administration; supervision; writing – review and editing. **Uri Weinberg:** Conceptualization; writing – review and editing; project administration; supervision. **Yoram Palti:** Conceptualization; supervision; project administration; writing – review and editing.

## FUNDING INFORMATION

This study was funded by Novocure Ltd.

## CONFLICT OF INTEREST STATEMENT

TK, THK, BK, CD, KWK, AV, SZR, HME, RFG, SC, LL, EZT, MS, and AH are employees of Novocure and hold company stocks. YB, TV, LK, BB, AKG, MG, and UW are employees of Novocure and hold company stocks and TTFields‐related patents assigned to Novocure. YP is the founder of Novocure and holds company stocks and TTFields‐related patents assigned to Novocure.

## ETHICS STATEMENT

All animal studies were approved by the Animal Experimentation Committee according to the Ministry of Health by the Israel National Council for Animal Experimentation and performed in accordance with guidelines and regulations for the care of laboratory animals.

## Supporting information


**Data S1.** Supporting Information.

**Table S1.** Sequencing coverage and quality statistics.

## Data Availability

The RNA‐seq data is available in the ArrayExpress database (http://www.ebi.ac.uk/arrayexpress) under accession number E‐MTAB‐15132 (https://www.ebi.ac.uk/biostudies/ArrayExpress/studies/E‐MTAB‐15132?key=a8635707‐9759‐40fd‐8d69‐c9953022571a). Other data that support the findings of this study are available from the corresponding authors upon request.
