# Supplementary material for: Efficacy and immunogenic effects of Tumor Treating Fields (TTFields) in preclinical models of pancreatic ductal adenocarcinoma, with and without gemcitabine/nab‐paclitaxel
Source: Int J Cancer. 2026 Feb 27;158(12):3091–100. doi: 10.1002/ijc.70408 (PMC13106910; doi:10.1002/ijc.70408)
Supplement: Supplementary file 1 — Data S1. Supporting Information. [file IJC-158-3091-s001.pdf]

Supplementary Materials

Efficacy and Immunogenic Effects of Tumor Treating Fields (TTFields) in Preclinical Models of Pancreatic Ductal Adenocarcinoma with and without Gemcitabine/Nab-paclitaxel

Short title: TTFields for the treatment of PDAC

Tal Kan, Tharwat Haj Khalil, Yiftah Barsheshet, Tali Voloshin, Lilach Koren, Bella Koltun, Cfir David, Kerem Wainer-Katsir, Avital Vorontsov, Boris Brant, Simona Zisman-Rozen, Hila M Ene, Roni Frechtel-Gerzi, Shay Cahal, Anat Klein-Goldberg, Lena Lifshitz, Efrat Zemer Tov, Mai Shai, Adi Haber, Moshe Giladi, Uri Weinberg, and Yoram Palti

Table of Contents

1. Supplementary Table S1 (available in a separate Excel file)
